# eIF5B increases ASAP1 expression to promote HCC proliferation and invasion

**DOI:** 10.18632/oncotarget.11469

**Published:** 2016-08-22

**Authors:** Zhen-guang Wang, Hao Zheng, Wei Gao, Jun Han, Jing-zhu Cao, Yuan Yang, Shuai Li, Rong Gao, Hui Liu, Ze-ya Pan, Si-yuan Fu, Fang-ming Gu, Hao Xing, Jun-sheng Ni, Hong-li Yan, Hao Ren, Wei-ping Zhou

**Affiliations:** ^1^ The Third Department of Hepatic Surgery, Eastern Hepatobiliary Surgery Hospital, Second Military Medical University, Shanghai 200433, China; ^2^ Department of Microbiology, Shanghai Key Laboratory of Medical Biodefense, Second Military Medical University, Shanghai 200433, China; ^3^ Department of Laboratory Medicine, Changhai Hospital, The Second Military Medical University, Shanghai 200433, China; ^4^ Department of Computer Science, Rensselaer Polytechnic Institute, Troy, NY 12180, USA; ^5^ Department of Endocrinology, Changhai Hospital, The Second Military Medical University, Shanghai 200433, China

**Keywords:** eIF5B, ASAP1, hepatocellular carcinoma, proliferation, invasion

## Abstract

Hepatocellular carcinoma (HCC) is the third most common cause of cancer-related death worldwide. Despite the therapeutic advances that have been achieved during the past decade, the molecular pathogenesis underlying HCC remains poorly understood. In this study, we discovered that increased expression eukaryotic translation initiation factor 5B (eIF5B) was significantly correlated with aggressive characteristics and associated with shorter recurrence-free survival (RFS) and overall survival (OS) in a large cohort. We also found that eIF5B promoted HCC cell proliferation and migration *in vitro* and *in vivo* partly through increasing ASAP1 expression. Our findings strongly suggested that eIF5B could promote HCC progression and be considered a prognostic biomarker for HCC.

## INTRODUCTION

Hepatocellular carcinoma (HCC) is the third most common cause of cancer-related death worldwide [1–3]. Despite recent therapeutic advances in the treatment of HCC, such as surgical resection, liver transplantation, and adjuvant therapy, the overall 5-year survival rate of patients with HCC remains poor [4, 5]. The molecular pathogenesis underlying HCC in human remains poorly understood. Therefore, it is necessary to elucidate the mechanism of hepatocarcinogenesis to provide useful information for the clinical management of HCC.

eIF5B is a eukaryotic translational GTPase that catalyzes ribosomal subunits, which join to form elongation-competent ribosomes [6, 7]. eIF5B is the mammalian ortholog of bacterial IF-2 and is important for the formation of the 30S initiation complex, stimulation of 50S association to form 70S complexes, and stabilization of the tRNA-Meti association with the ribosome [8–11]. eIF5B functions in 60S ribosome subunit joining during canonical translation [12] and is involved in pre-40S ribosome subunit proofing [13, 14]. eIF5B is also required for the translation of a few viral and specialized mRNAs and contributes to supporting or stabilizing the tRNA-Meti association, including under specific conditions where phosphorylation of the eIF-2 subunit eIF-2α is also observed [15–18]. However, the function and precise mechanisms of eIF5B in HCC are unknown and require further investigation.

In the present study, we demonstrated that eIF5B expression is increased in HCC cells and tumor tissues and that this increased expression of eIF5B is associated with poor outcome in patients with HCC. Moreover, the increased expression of eIF5B dramatically promoted HCC proliferation and invasion both *in vitro* and *in vivo* largely by increasing ASAP1 expression. These findings suggest that eIF5B promotes HCC development and progression and may be useful for future HCC therapy.

## RESULTS

### eIF5B expression is increased in human HCC cell lines

**Figure 1:**
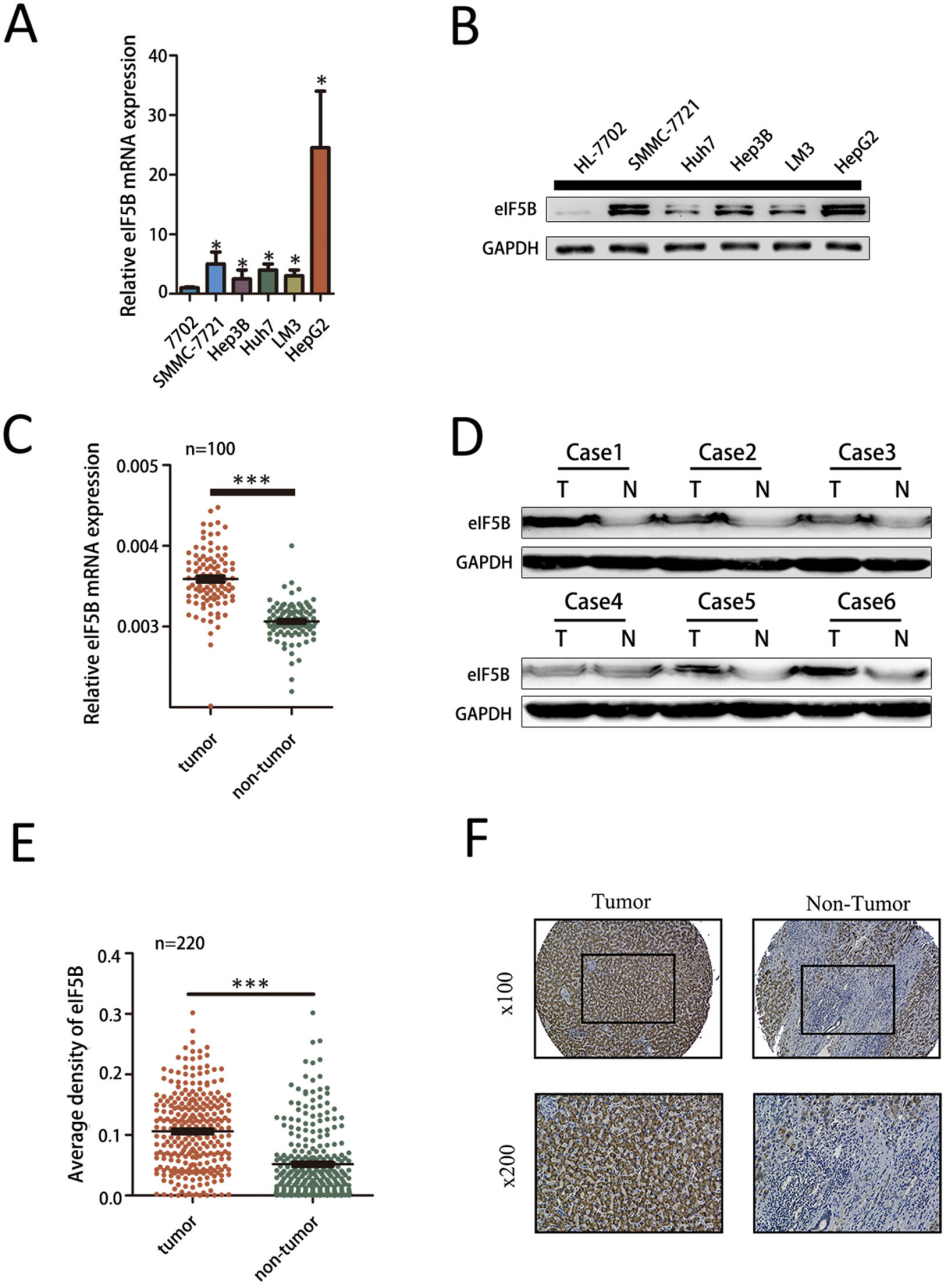
eIF5B expression is increased in human HCC cell lines **A-B.** eIF5B mRNA and protein expression is increased in several HCC cells compared with the normal liver cell line (P<0.05). **C.** eIF5B mRNA expression levels in 100 paired HCC and adjacent non-tumor tissues were evaluated by quantitative real-time polymerase chain reaction. **D.** Western blot analysis of the eIF5B protein level in tumor tissues (T) and paired adjacent non-tumor tissues (N) from 6 patients with HCC. **E.** Relative IHC staining of eIF5B expression in paired HCC tissue samples (n=220). eIF5B expression was significantly increased in tumors compared with the corresponding adjacent non-tumor liver tissues. **F.** Representative IHC image of tumor and peri-tumor tissues. No staining is denoted by blue, weak staining by light brown, moderate staining by brown, and strong staining by dark brown. (*P<0.05; ***P<0.001).

To clarify the underlying function of eIF5B in HCC progression, we first examined eIF5B mRNA expression levels in 5 HCC cell lines and 1 normal liver cell line (HL-7702) using qRT-PCR. We found that eIF5B expression was significantly increased in the HCC cell lines compared with the normal liver cell line (Figure 1A). Similar results were also observed by western blot analysis (Figure 1B). To further study eIF5B expression in tumor tissues, we examined a panel of paired tumor and normal primary tissue specimens that were collected from patients with HCC (n=100). The eIF5B transcript levels were much higher in the tumor tissues compared with the non-tumor tissues (Figure 1C). We then examined eIF5B protein expression in 6 paired HCC tumor and normal primary tissues and found that eIF5B expression is higher in tumor tissues than in normal primary tissues (Figure 1D). Immunohistochemical (IHC) analysis of eIF5B expression was performed using HCC tissue microarrays (TMAs) containing 220 paired HCC samples. As shown in Figure 1E, the staining density of the eIF5B protein in the tumor group was stronger than that observed in the peri-tumor group (P<0.001). Representative IHC staining is shown in Figure 1F.

### Increased eIF5B expression predicts aggressive clinic pathological characteristics and poor prognosis in patients with HCC

**Figure 2:**
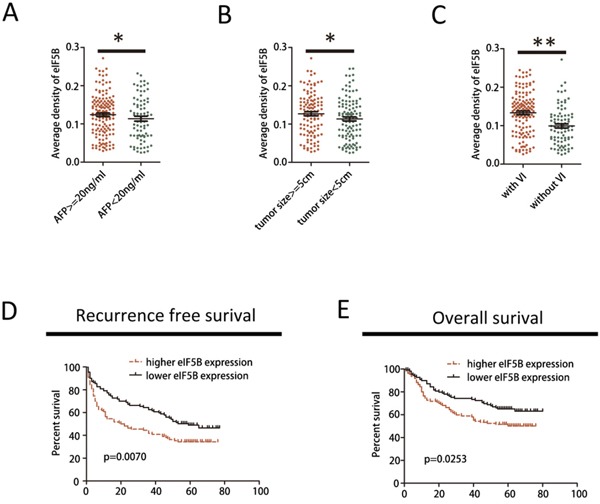
eIF5B up-regulation predicts aggressive clinicopathological characteristics and poor prognosis in patients with HCC **A-C.** Expression patterns of eIF5B in patients with HCC with high vs. low AFP levels, large vs. small tumors, and positive vs. negative vessel invasion (VI). **D-E.** Kaplan-Meier survival curves of recurrence-free survival (RFS) and overall survival (OS) in the cohort. Patients were assigned into one of two subgroups according to the median expression level of eIF5B (*P<0.05; **P<0.01).

To further investigate the clinical significance of eIF5B expression in the development and progression of HCC, all 220 patients with HCC were analyzed. As shown in Figure 2A-2C, increased eIF5B expression was observed in tumors with a higher AFP level, larger tumors and vascular invasion (VI) (Figure 2A–2C) compared with tumors with a lower AFP level, smaller tumors and without VI, which has been reported to be an independent early recurrence factor after primary resection in patients with HCC [19]. Then, the 220 patients with HCC were divided into 2 groups based on their overall eIF5B expression level: a high eIF5B expression group (n=110) and a low eIF5B expression group (n=110) (Table 1). Subsequently, Kaplan-Meier analysis revealed that patients with high eIF5B expression developed more frequent recurrence (Figure 1D) and poorer survival (Figure 1E) after hepatectomy compared with patients with low eIF5B expression. Collectively, these results indicated that eIF5B might be a critical oncogene that promotes HCC development and progression.

**Table 1:** Clinical characteristics of 220 HCC patients according to eIF5B expression level

Feature	eIF5B		χ^2^	p-value
	Low	High^#^		
**All cases**				
**Age, y**			0.948	0.330
**>55**	38	45		
**≤55**	72	65		
**Gender**			0.768	0.381
**Male**	98	101		
**Female**	12	9		
**HBsAg**			6.370	0.012
**Positive**	94	105		
**Negative**	16	5		
**HBeAg**			1.592	0.207
**Positive**	22	15		
**Negative**	88	95		
**AFP, μg/L**			1.986	0.159
**Positive**	66	76		
**Negative**	44	34		
**Tumor size, cm**			1.819	0.177
**>5**	51	61		
**≤5**	59	49		
**Tumor number**			0.698	0.403
**Single**	90	85		
**Multiple**	20	25		
**Vascular invasion**			24.958	0.000
**Present**	50	86		
**Absent**	60	24		
**Tumor Differentiation**			11.859	0.001
**I-II**	16	2		
**III-IV**	94	108		

# The median expression level was used as the cut-off. Low eIF5B expression in each of the 110 patients was defined as a value below the 50th percentile. High eIF5B expression in each of the 110 patients was defined as a value above the 50th percentile.

* For analysis of correlation between the expressions levels of eIF5B and clinical features, Pearson chi-square tests were used. Results were considered statistically significant at p<0.05.

### eIF5B induces metastasis and proliferation *in vitro*

**Figure 3:**
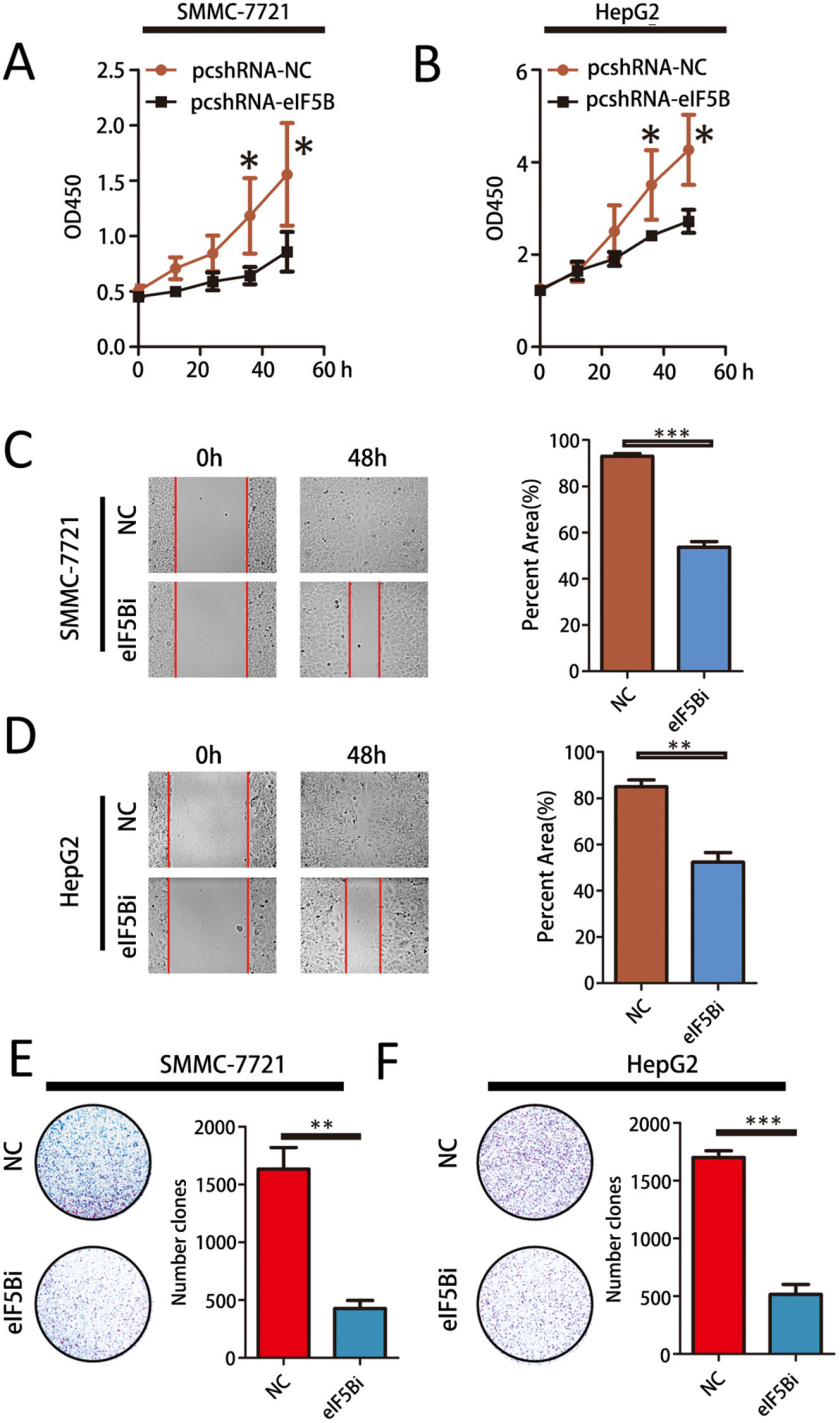
eIF5B promotes metastasis and proliferation *in vitro* **A-B.** Cell Counting Kit-8 (CCK8) cell proliferation assay. Parental cells transfected with empty vector were used as a control. A t-test was performed to evaluate statistically significant differences between the groups. **C-D.** The migratory properties of eIF5B knockdown cells compared with control cells were analyzed by scratch wound healing assays. Representative results are shown. Magnification: 100x. **E-F.** Representative images showing colony formation by the different stable cell lines (left panels). The colony formation rate (%) was calculated by dividing the colony number by 200 plated cells (right panels). The data represent the means±SEM from three independent experiments (*P<0.05; **P<0.01; ***P<0.001).

To investigate the biological significance of eIF5B-induced proliferation and invasion *in vitro*, we examined the effects of eIF5B loss-of-function and gain-of-function on cell phenotypes. As eIF5B expression is higher in SMMC-7721 and HepG2 cells and lower in HCCLM3 and Huh7 cells, we developed SMMC-7721 and HepG2 cell lines in which eIF5B expression was stably knocked down (Supplementary Figure S1A-B, S1E-S2F) and HCCLM3 and Huh7 cell lines in which eIF5B expression was stably overexpressed (Supplementary Figure S1C-D, S1G-S2H). We then observed the effect of eIF5B expression on cell proliferation, invasion, and colony formation. Suppressing cellular eIF5B expression not only inhibited proliferation (Figure 3A and 3B) but also reduced invasion and colony formation in SMMC-7721 and HepG2 cells compared with the negative control (NC) cells (Figure 3C–3F). However, we found that eIF5B overexpression could promote cell proliferation (Supplementary Figure S2A and S2B), invasion and colony formation in HCCLM3 and Huh7 cell lines (Supplementary Figure S2C-S2F).

### eIF5B knockdown inhibited tumor growth *in vivo*

**Figure 4:**
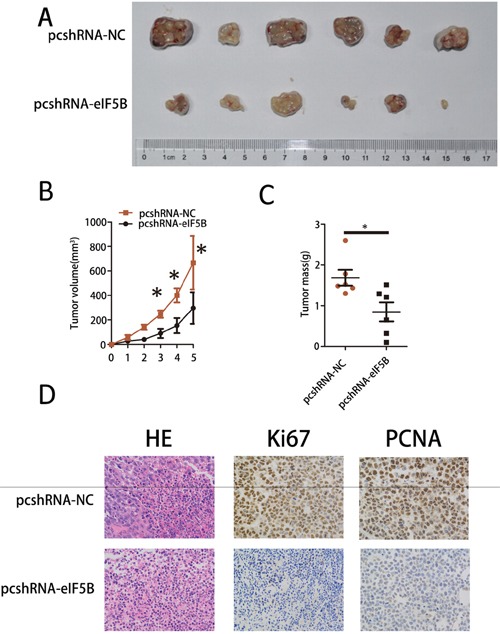
eIF5B knockdown inhibited tumor growth *in vivo* **A.** Images, **B.** tumor growth and **C.** weights of xenografts established by subcutaneous transplantation with pcshRNA-eIF5B-transfected and control-transfected SMMC-7721 cells 5 weeks after cell injection (n=6). **D.** H&E-stained paraffin-embedded sections obtained from xenografts. IHC staining shows that Ki67 and PCNA expression was enhanced in the pcshRNA-NC group compared with the pcshRNA-eIF5B group. Original magnification, 400× (*P<0.05).

To determine the effects of eIF5B on tumorigenesis *in vivo*, we subcutaneously injected SMMC-7721 cells that had been stably transfected with pcshRNA-NC or pcshRNA-eIF5B into nude mice for xenotransplantation. Mice injected with pcshRNA-eIF5B-transfected cells (Figure 4A, pcshRNA-eIF5B) showed significantly decreased tumor growth compared with animals injected with pcshRNA-NC-transfected cells (Figure 4A, pcshRNA-NC). As assessed by measurements of tumor volume and mass, eIF5B knockdown significantly decreased overall tumor growth (Figure 4B and 4C). Two cellular proliferation antigens, Ki67 and PCNA, were detected by IHC in the tumor tissues from xenografts; the expression of both of these antigens was significantly stronger in xenografts of pcshRNA-NC-transfected cells than in xenografts of pcshRNA eIF5B-transfected cells (Figure 4D).

### eIF5B increases ASAP1 expression in HCC cell lines

**Figure 5:**
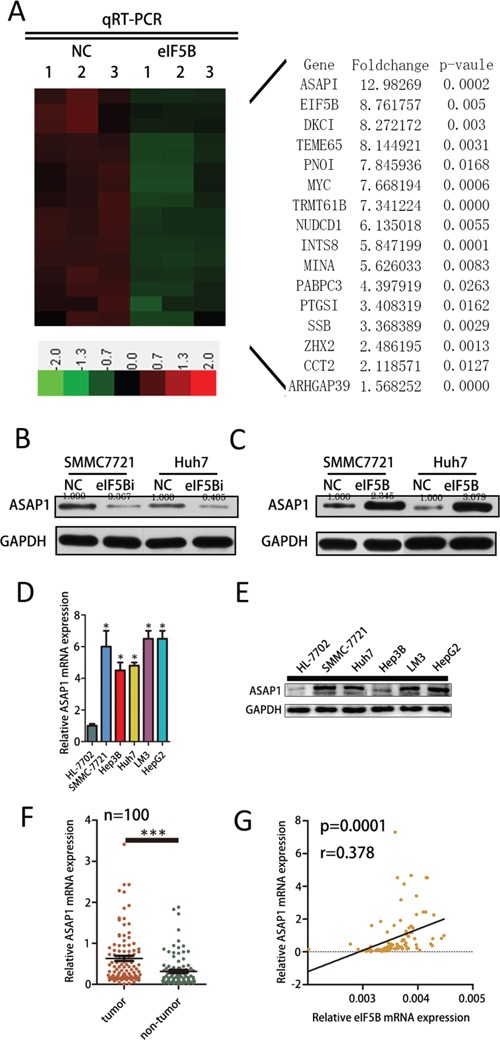
eIF5B increases ASAP1 expression in HCC cell lines **A.** Hierarchical clustering analysis of genes whose expression decreased in the eIF5B cells. **B.** Western blot analysis of ASAP1 protein levels in NC and eIF5B knockdown SMMC-7721 and Huh7 cell lines. **C.** Western blot analysis of ASAP1 protein levels in NC and eIF5B overexpression SMMC-7721 and Huh7 cell lines. **D.** ASAP1 mRNA levels in the HL-7702 cell line and several HCC cell lines. **E.** Western blot analysis of ASAP1 protein levels in the HL-7702 cell line and in several HCC cell lines. **F.** ASAP1 mRNA expression levels in 100 paired HCC and adjacent non-tumor tissues as evaluated by quantitative real-time polymerase chain reaction (P<0.05). **G.** The correlation between the eIF5B transcript level and ASAP1 mRNA level was measured in the same set of tissues (*P<0.05; ***P<0.001).

Next, we explored the mechanisms leading to the potent effect of eIF5B on cell proliferation and invasion. We analyzed eIF5B expression in The Cancer Genome Atlas (TCGA) database and found that the co-expression of 1156 genes with eIF5B was closely correlated (correlation>0.3). Of these genes, 811 genes were positively correlated with eIF5B expression, and 345 genes were negatively correlated with eIF5B expression. Supplementary Figure S3A shows the main pathway associated with the co-expressed genes that positively correlated with eIF5B expression (correlation>0.3) as determined by KEGG pathway analysis (http://www.genome.jp/kegg/). We used PCR to verify the top 50 co-expressed genes that positively correlated with eIF5B and found that the expression of 16 of these genes was decreased in eIF5B knockdown cell lines compared with the control (P<0.05) (Figure 5A). Notably, we found that ASAP1 is highly co-expressed with eIF5B (Figure 5A). Considering the function of ASAP1 in proliferation and metastasis [20, 21], we thus reasoned that eIF5B might promote proliferation and metastasis through increasing ASAP1 expression. We found that reducing eIF5B expression decreased ASAP1 protein levels in the SMMC-7721 and Huh7 cell lines (Figure 5B) and that eIF5B overexpression increased ASAP1 protein levels in the SMMC-7721 and Huh7 cell lines (Figure 5C). Moreover, we discovered that ASAP1 mRNA and protein expression levels were decreased in the tumor tissues from xenografts of eIF5B knockdown SMMC-7721 cells (Supplementary Figure S3B and S3C). ASAP1 mRNA and protein levels are lower in HL-7702 cells compared with the HCC cell lines (Figure 5D and 5E). To confirm the effect of eIF5B on ASAP1 expression in human HCC tissues, we measured the ASAP1 mRNA level in the same set of 100 pairs of HCC tissues shown in Figure 5F and found that ASAP1 mRNA expression was significantly higher in the tumor tissues than in the non-tumor tissues. Moreover, the ASAP1 mRNA level was correlated with the eIF5B transcript level in the tumor tissues (Figure 5G). These clinical data demonstrated that eIF5B could increase ASAP1 expression.

### eIF5B requires ASAP1 signaling to promote HCC proliferation and invasion

**Figure 6:**
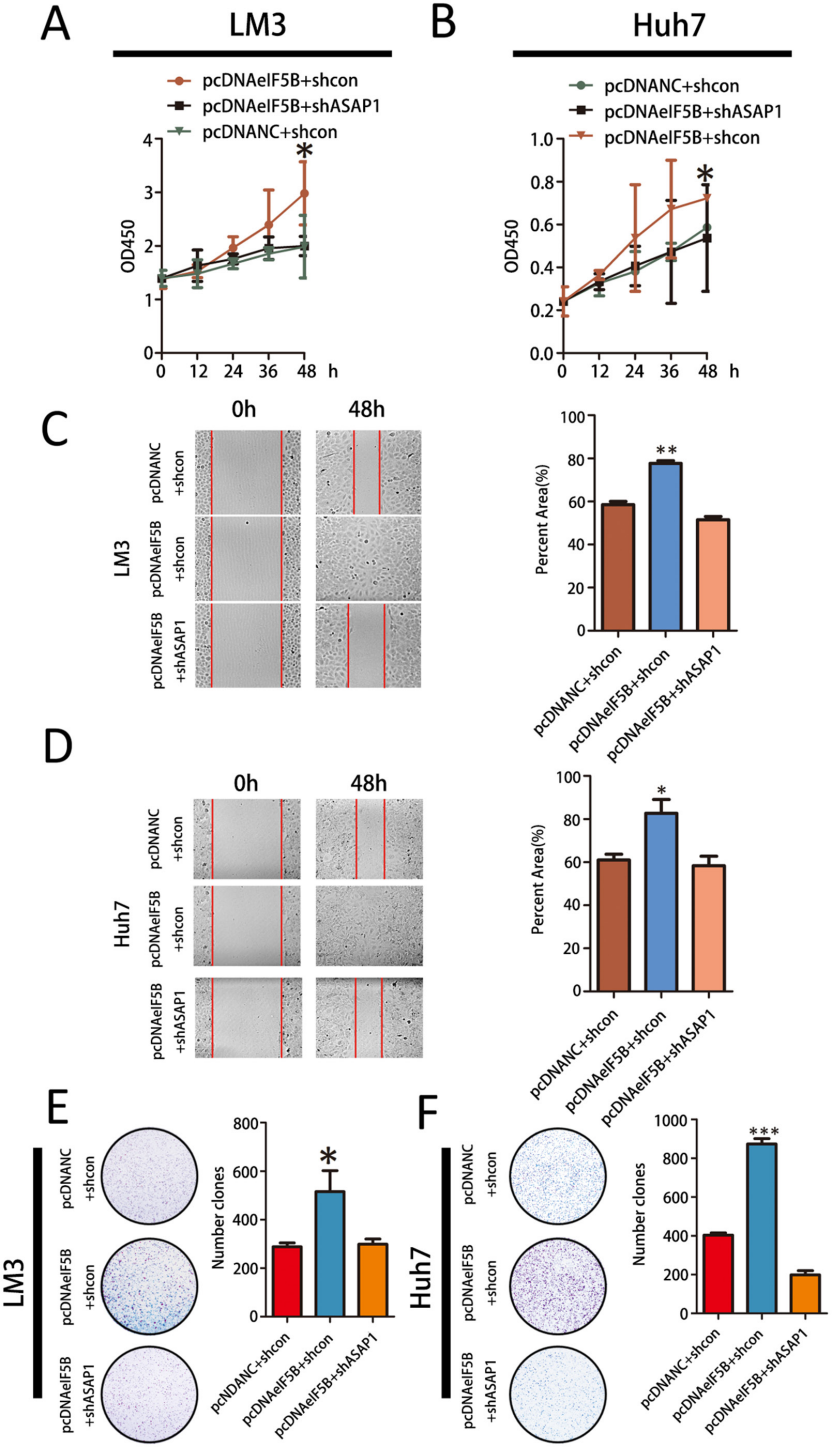
eIF5B requires ASAP1 signaling to promote HCC proliferation and invasion **A-B.** Cell Counting Kit-8 (CCK8) cell proliferation assay. Parental cells transfected with empty vector were used as controls. A t-test was performed to evaluate statistically significant differences between the two groups. **C-D.** The migratory properties of eIF5B overexpression cells compared with control cells were analyzed by scratch wound healing assays. Representative results are shown. Magnification: 100x. **E-F.** Representative images showing colony formation by the different stable cell lines (left panels). The colony formation rate (%) was calculated by dividing the colony number by 200 plated cells (right panels). The data represent the means±SEM from three independent experiments (*P<0.05; **P<0.01; ***P<0.001).

To determine the effect of ASAP1 on eIF5B function, we first overexpressed ASAP1 in eIF5B knockdown cell lines. This overexpression had no significant effect on eIF5B expression in SMMC-7721 cell lines (Supplementary Figure S4A-S4B). Then, we knocked down ASAP1 expression in eIF5B overexpression LM3 and Huh7 cells (Supplementary Figure S4C-S4F) and measured cell proliferation and invasion abilities. ASAP1 knockdown abolished the effects of eIF5B on these parameters (Figure 6A–6F). These data demonstrated that eIF5B enhanced the proliferation and invasion potential of HCC cells and that this effect required ASAP1.

## DISCUSSION

In the present study, we reported that eIF5B expression was significantly increased in HCC tissues compared with that in adjacent normal liver tissue. The increase in eIF5B expression was also frequently observed in both HCC cells and fresh tissue samples. Additionally, we investigated the prognostic effect of eIF5B expression on the overall survival (OS) of patients with HCC. Kaplan-Meier curve assessment showed that high eIF5B expression levels predicated poor survival for patients with HCC. Taken together, our data indicated that eIF5B expression was associated with HCC progression and might serve as an independent prognostic marker for the OS of patients with HCC.

We next explored the possible function of eIF5B in HCC progression. The results showed that suppressing eIF5B expression inhibited HCC cell proliferation, motility and invasion *in vitro*. Therefore, we sought to determine whether eIF5B knockdown could suppress tumor growth *in vivo*. We found that the eIF5B knockdown obviously decreased subcutaneous tumor growth. Thus, the above-described results provide evidence that eIF5B can functionally promote HCC proliferation and invasion *in vitro* and *in vivo*.

To explore the mechanism by which eIF5B contributes to HCC proliferation and invasion, we analyzed TCGA database and found that the correlation coefficient for eIF5B and ASAP1 co-expression was high (correlation=0.515). Additionally, eIF5B was found to promote HCC proliferation and metastasis through increasing ASAP1 expression. ASAP1 is a multi-domain protein that promotes cell invasion and motility [22–27]. This protein contains proline-rich SH3-binding motifs and an SH3 domain that binds with adaptor and signaling proteins such asc-src and Crk [22, 28], CD2AP/CMS [22], cortactin [29], CIN85 [30], CrkL [31], POB1 and paxillin [23], FAK [32] and Pyk2 [33, 34]. ASAP1 is able to bend membranes for EGFR trafficking due to a BAR domain at its N-terminus [35]. Furthermore, ASAP1 is capable of binding to phosphoinositide phospholipids through its pleckstrin homology domain, which promotes Arf-GTP activity [36]. ASAP1 has been shown to be overexpressed and enhanced in several malignancies and is functionally correlated with breast cancer metastasis [27, 29, 37, 38]. ASAP1 has been reported to be a prognostic marker for patients with laryngeal squalors cell carcinoma (LSCC) [20] and patients with epithelial ovarian cancer (EOC) [21]. Here, we found that eIF5B could promote HCC cell proliferation and metastasis through increasing ASAP1 expression; however, the detailed mechanism by which eIF5B increases ASAP1 expression remains unknown. Eukaryotic translation initiation is a highly regulated process involving multiple steps from 43S pre-initiation complex (PIC) assembly to ribosomal subunit joining. A previous study reported that the interactions between eIF1A and eIF5B are being continuously rearranged during translation initiation [39]. Because eIF1A and eIF5B bind to adjacent sites on the ribosome, their effective concentrations with respect to each other would also increase, further stabilizing their interaction. It has been reported that eIF5B is the mammalian ortholog of bacterial IF2, which is important for the formation of the 30S initiation complex, stimulates 50S association to form 70S complexes, and can contribute to stabilizing the tRNA-Meti association with the ribosome [8–11]. We hypothesize that eIF5B may function as a translation initiation complex that indirectly promotes ASAP1 transcription. This hypothesis requires verification by further experiments.

In summary, our study demonstrates that increased eIF5B expression is significantly associated with HCC progression and poor survival of patients with HCC. All of the functional experiments also confirm the function of eIF5B as a tumor driver in HCC progression. The preferential increase in eIF5B expression in patients with HCC, together with HCC proliferation and invasion, suggests that eIF5B might be a significant biomarker for HCC.

## MATERIALS AND METHODS

### Tissue microarray construction and samples

TMA slides were constructed in collaboration with Shanghai Biochip Company (Shanghai, China) as described previously [40, 41]. The detailed clinicopathologic characteristics of the patients are listed in Table 1. All patients were followed until August 2012, according to a previously reported uniform guideline, with a median observation time of 60 months. The time of the surgery was used to calculate the time to a given event. OS was defined as the interval between surgery and the date of death. Recurrence-free survival (RFS) was defined as the interval between surgery and the date of recurrence. OS and RFS were censored at the last follow-up visit (August 31, 2012) for patients without death or recurrence. Micro-metastases were defined as tumors adjacent to the border of the main tumor and were only observed under a microscope. Tumor differentiation was graded according to the Edmondson–Steiner grading system [42]. Tumor stage was defined according to the Barcelona Clinic Liver Cancer (BCLC) staging system [43].

An additional 106 patients with HCC were recruited between January 1 and September 30, 2010, and their resected samples were subjected to RNA extraction for verification by quantitative RT-PCR (100 samples) and western blot (6 samples).

### Cell culture

Human HCC cell lines (SMMC-7721, HCC-LM3, Hep3B, Huh7, HepG2 and HL-7702) were purchased from the Shanghai Institute of Life Sciences Cell Resource Center (Shanghai, China). All cell lines were cultured in DMEM (HyClone, CA, USA) supplemented with 10 % fetal bovine serum (FBS) and 1 % penicillin/streptomycin (Gibco, CA, USA). All cell cultures were maintained at 37°C in a humidified atmosphere with 5 % CO_2_.

### Reverse transcription and real-time polymerase chain reaction (RT-PCR) analysis

Total RNA was extracted from frozen tumor specimens and cell lines using TRIzol reagent (Invitrogen, CA, USA). The extracted total RNA was then reverse-transcribed using random primers and a M-MLV Reverse Transcriptase Kit (Invitrogen, CA, USA) according to the manufacturer's instructions. The expression of eIF5B and associated encoding genes was measured using SYBR real-time PCR (qPCR) (TaKaRa Bio, Otsu, Japan) according to the manufacturer's instructions. The PCR conditions were 95°C for 5 min, followed by 40 cycles at 95°C for 15 s, 58°C for 15 s and 72°C for 20 s. GAPDH was used as an internal standard. The sequences of the primers and probes used in the qPCR reactions are listed in Supplementary Table S1.

### Colony formation assay

The cells were collected and placed in 1-cm plates (100 cells per plate). After the cells were cultured for 14 days, the surviving colonies were fixed in methanol, washed two times with phosphate-buffered saline, dried, stained with liquid crystal violet (Sigma-Aldrich, Dorset), and counted. The experiments were repeated three times.

### Cell counting kit-8 (CCK8) assay

Cells were transfected with eIF5B shRNA or lenti-vector and were cultured for 12, 24, 48 and 72 h. Wells containing only culture medium served as blanks. At each time point, the supernatant was removed, and 100 μL of DMEM containing 10 μL of CCK8 was added to each well for additional 2 h incubation at 37°C. The absorbance was recorded at 450 nm. All of the experiments were independently repeated at least three times.

### Wound healing assays

For the wound healing assays, monolayer cells plated in 12-well plates were wounded by scraping with a 200-μl plastic pipette tip and then rinsed several times with medium to remove the floating cells. The wound healing process was monitored with an inverted light microscope (Olympus).

### Subcutaneous xenografts

Subcutaneous xenografts were established in the bilateral flanks of athymic nude mice using SMMC-7721 cells (5×10^6^) infected with either pcDNA-NC or pcDNAsheIF5B at a MOI of 20. The tumor volume was calculated using the following formula: larger diameter× (smaller diameter)^2^/2. All of the experiments were repeated three times.

### Western blot analysis

Total cell and tissues lysates were prepared in 1× sodium dodecyl sulfate buffer. Identical quantities of proteins were separated by sodium dodecyl sulfate-polyacrylamide gel electrophoresis and transferred onto nitrocellulose filter membranes. After the blots were incubated with antibodies specific for eIF5B (Abcam, CA), ASAP1 (Abcam, CA) or GAPDH (Abcam, CA), they were incubated with IRDye 800-conjugated goat anti-rabbit IgG and IRDye 700-conjugated goat anti-mouse IgG, and signals were detected using an Odyssey infrared scanner (Li-Cor). GAPDH was used as a loading control for western blots.

### Immunohistochemical (IHC) tissue section and tissue microarray analyses

Paraffin-embedded tissue sections and TMAs underwent IHC analyses. Briefly, the slides were probed with primary antibodies specific for the following proteins: eIF5B, PCNA, and Ki67. Anti-rabbit or anti-mouse horseradish peroxidase-conjugated secondary antibodies (Santa Cruz Biotechnology) were applied. Finally, the slides were stained with diaminobenzidine (DAB) colorimetric reagent solution from Dako (Carpinteria, CA) followed by hematoxylin counterstaining (Sigma Chemical Co). TMA analysis was performed by scanning the slides with an Aperio ScanScope GL, and Aperio ImageScope software (Aperio Technologies, Vista, CA) was used to assess the scanned images based on the percentage of positively stained cells and staining intensity. The expression levels of eIF5B in all clinical samples were quantified, and the ratio of the eIF5B expression level between each tumor/peri-tumor pair was calculated.

### Statistical analyses

Statistical analyses were performed using SPSS 18.0 and GraphPad Prism 5.0 software. Numerical data are presented as the means and standard errors. Differences between proportions were evaluated using a paired Student's t-test. P values≤0.05 were considered statistically significant. Each experiment was repeated at least three times.

## SUPPLEMENTARY FIGURES AND TABLE




