# Supplementary material for: eIF5B increases ASAP1 expression to promote HCC proliferation and invasion
Source: Oncotarget. 2016 Aug 22;7(38):62327–39. doi: 10.18632/oncotarget.11469 (PMC5308730; doi:10.18632/oncotarget.11469)
Supplement: Supplementary file 1 [file oncotarget-07-62327-s001.pdf]

eIF5B increases ASAP1 expression to promote HCC proliferation and invasion

SUPPLEMENTARY FIGURES AND TABLE

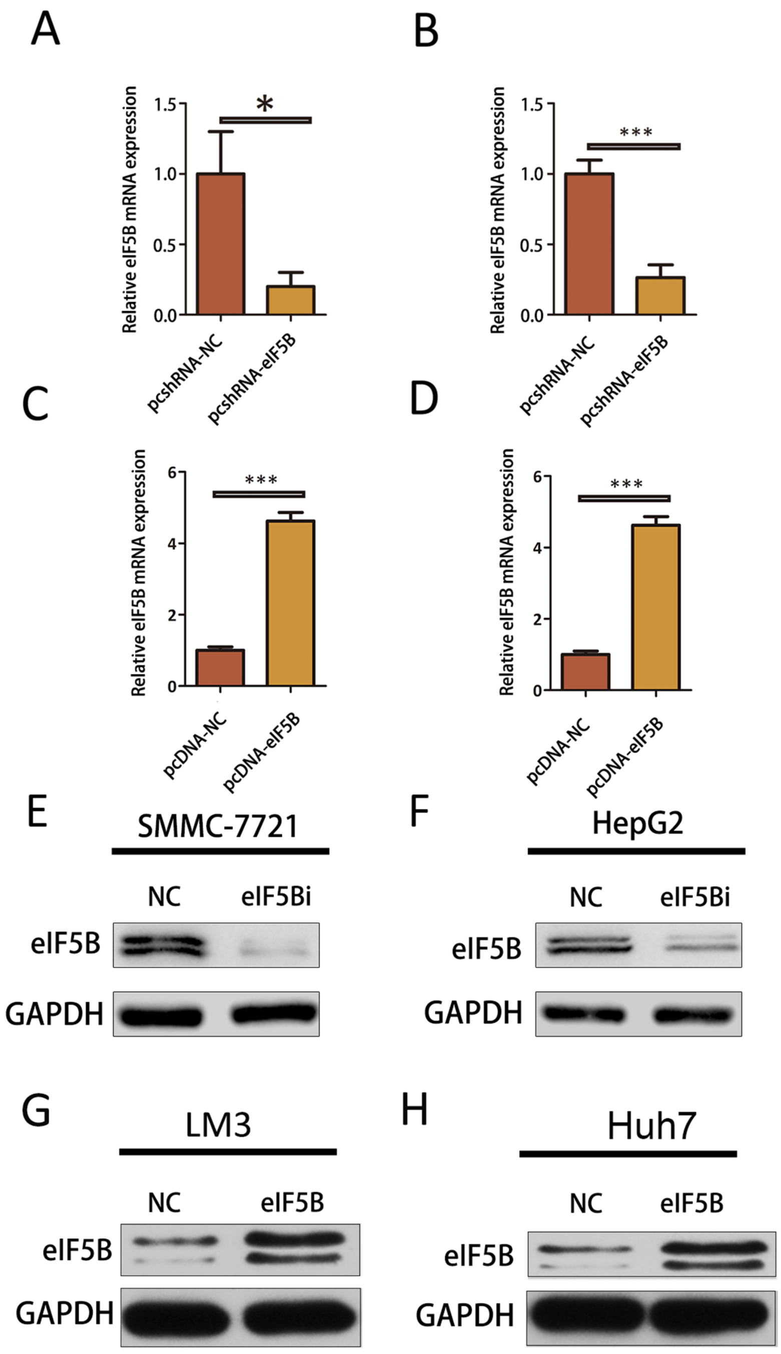

Supplementary Figure S1: eIF5B is stably knocked down in SMMC-7721 and HepG2 cells and overexpressed in LM3 and Huh7 cells. qRT-PCR and Western blotting were performed to assess the knockdown of eIF5B in SMMC-7721 cells **A** and **E.** and HepG2 cells **B** and **F.** and the overexpression of eIF5B in LM3 cells **C** and **G.** and Huh7 cells **D** and **H.** (*P<0.05; ***P<0.001).

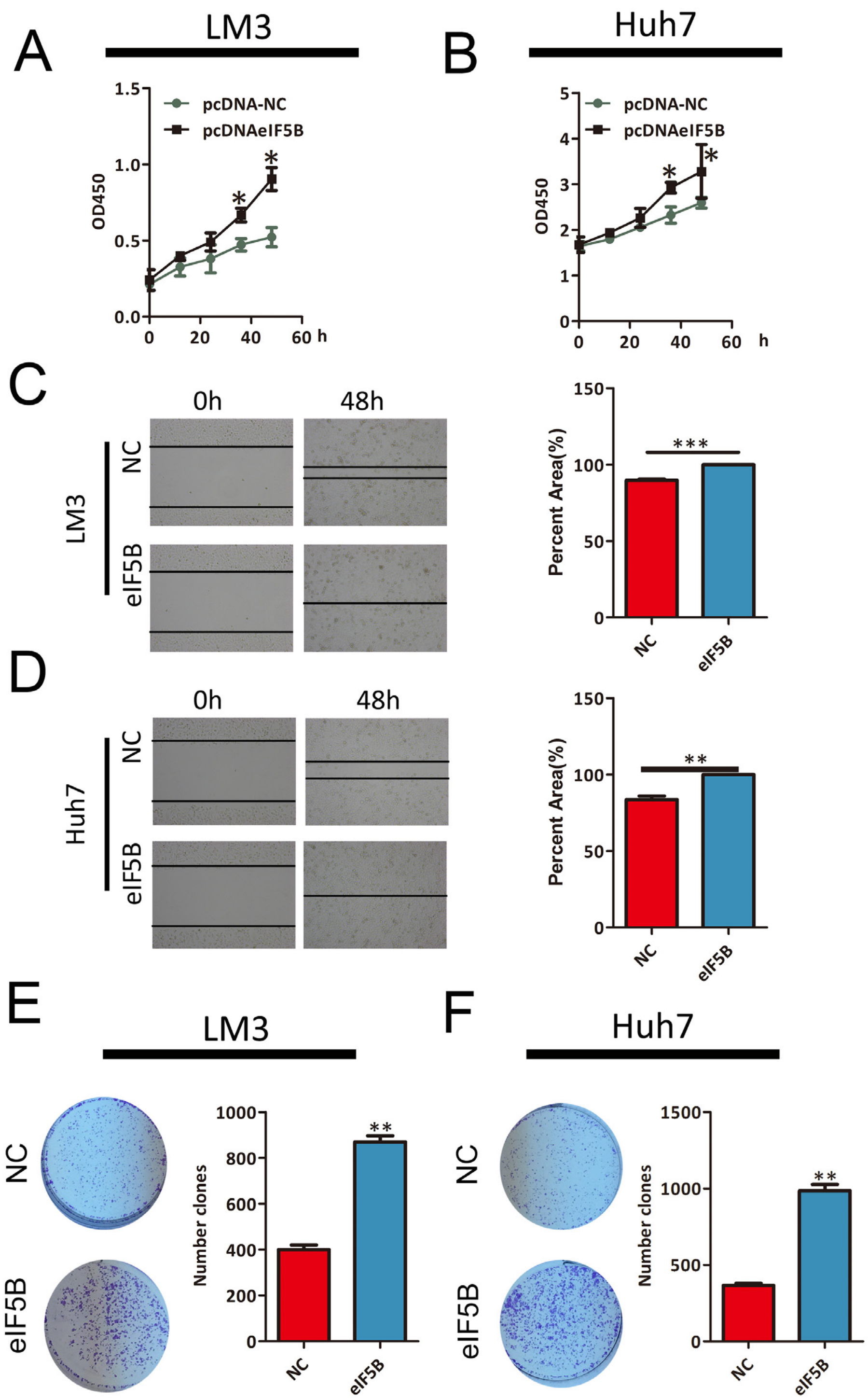

Supplementary Figure S2: eIF5B induces metastasis and proliferation in vitro. A-B. Cell Counting Kit-8 (CCK8) cell proliferation assay. Parental cells transfected with empty vector were used as controls. A t-test was performed to evaluate statistically significant differences between the groups. **C-D.** The migratory properties of eIF5B overexpressing cells compared with control cells were analyzed by scratch wound healing assays. Representative results are shown. Magnification: 100x. **E-F.** Representative images showing colony formation by the different stable cell lines (left panels). The colony formation rate (%) was calculated by dividing the colony number by 200 plated cells (right panels). The data represent the means±SEM from three independent experiments (*P<0.05;**P<0.01; ***P<0.001).

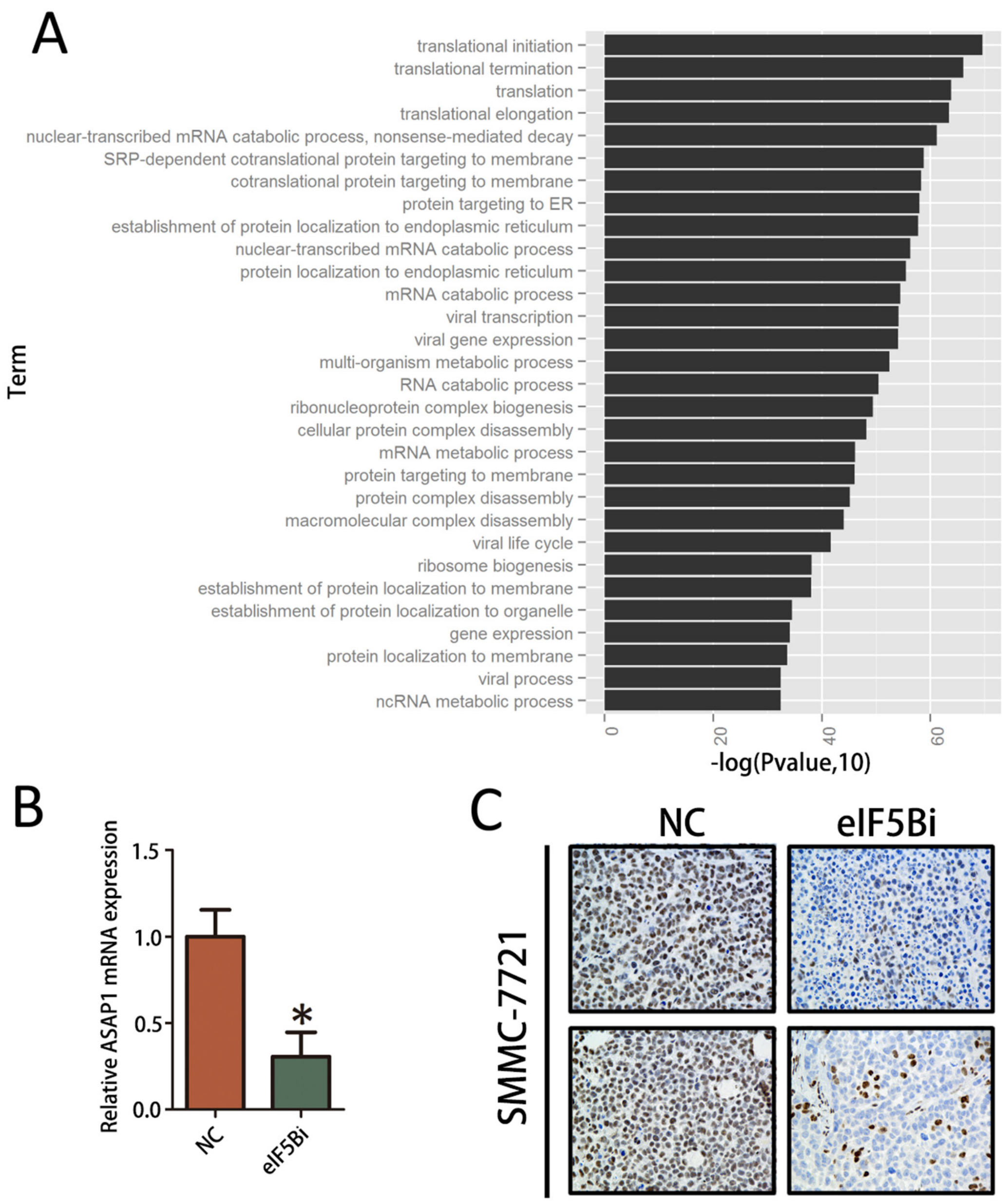

Supplementary Figure S3: ASAP1 expression is positively associated with eIF5B expression in mice. A. KEGG pathway analysis of the genes with positive co-expression (correlation>0.3) with eIF5B. **B.** Relative ASAP1 mRNA levels in tumors from mice(*P<0.05). **C.** Immunohistochemistry of ASAP1in tumors from (B).

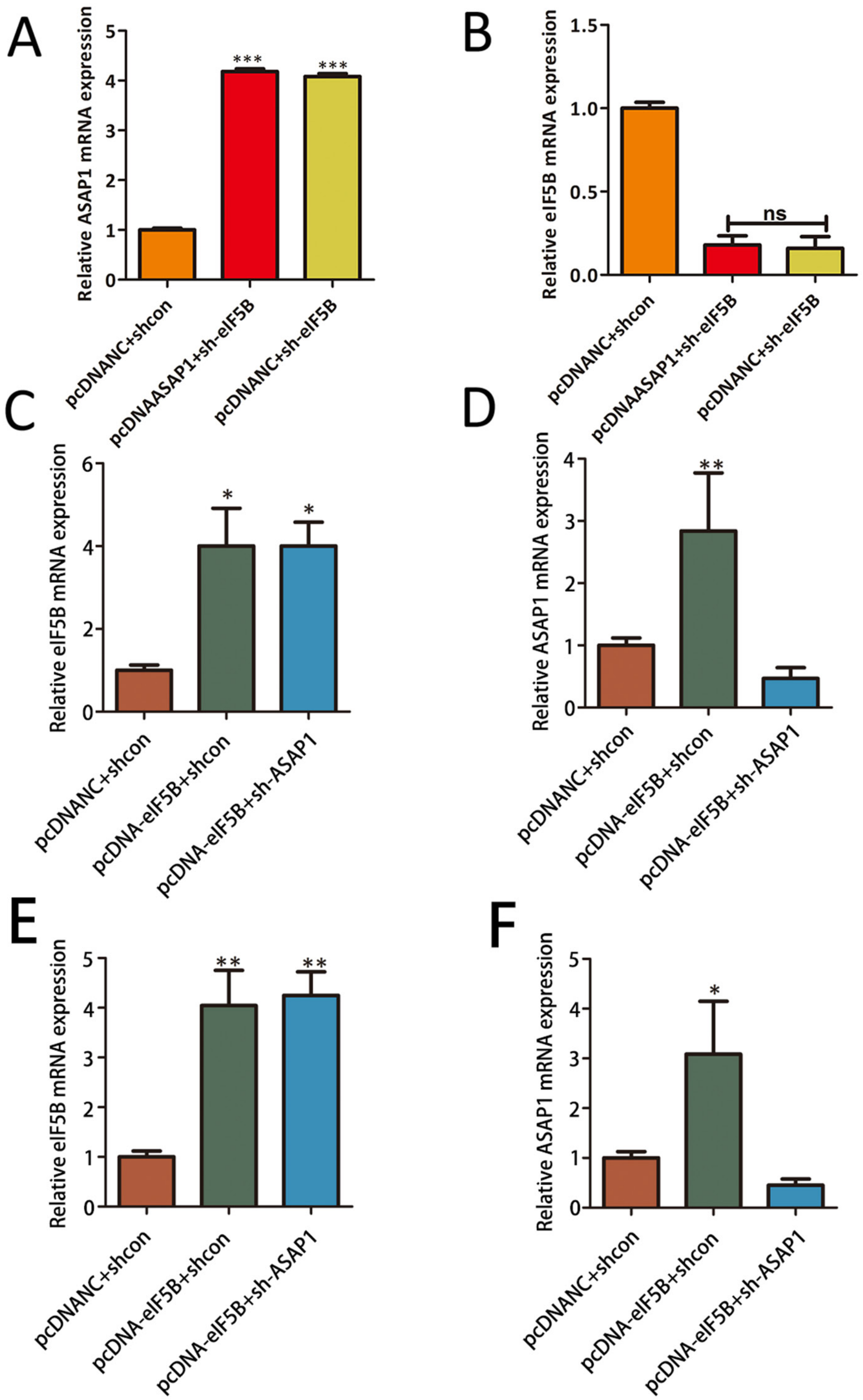

Supplementary Figure S4: ASAP1 expression is positively associated with eIF5Bexpression in LM3 and Huh7 cells. **A.** and **B.** The mRNA levels of ASAP1 and eIF5B in SMMC-7721 cells transfected with pcDNANC+shcon, pcDNAASAP1+sh-eIF5B, or pcDNANC+sh-eIF5B as detected by qRT-PCR. **C.** and **D.** The mRNA levels of eIF5B and ASAP1 in LM3 cells transfected with pcDNANC+shcon, pcDNAeIF5B+shcon, or pcDNAeIF5B+sh-ASAP1 as detected by qRT-PCR. **E.** and **F.** The mRNA levels of eIF5B and ASAP1 in Huh7 and SMMC-7721 cells transfected with pcDNANC+shcon, pcDNAeIF5B+shcon, or pcDNAeIF5B+sh-ASAP1 as detected by qRT-PCR(*P<0.05;**P<0.01; ***P<0.001).

Supplementary Table S1: Primer sequences for real-time PCR

See Supplementary File 1
