# Supplementary material for: eIF5B increases ASAP1 expression to promote HCC proliferation and invasion
Source: Oncotarget. 2016 Aug 22;7(38):62327–39. doi: 10.18632/oncotarget.11469 (PMC5308730; doi:10.18632/oncotarget.11469)
Supplement: Supplementary file 2 [file oncotarget-07-62327-s002.doc]

**Table s1** Primer sequences for real-time PCR

| [e](http://probes.pw.usda.gov/batchprimer3/batch_primers/58.32.217.78_1449208657/58.32.217.78_14492086571.html)IF5B forward | AGAGAGAAGCCAGAGCCAGA |
| --- | --- |
| eIF5B reverse | AATCATCCAATCCAGCATCC |
| GAPDH forward  GAPDH reverse | AGGTGAAGGTCGGAGTCAAC  CGCTCCTGGAAGATGGTGAT |
| [ASAP1](http://probes.pw.usda.gov/batchprimer3/batch_primers/58.32.217.78_1449208657/58.32.217.78_14492086571.html) forward | GGCAGAAGATGAGCAGGATT |
| ASAP1 reverse | TCCAAAGACTGAATGCGAGA |
| [ARFGEF1](http://probes.pw.usda.gov/batchprimer3/batch_primers/58.32.217.78_1449208657/58.32.217.78_14492086572.html) forward | CCCACCTCCATCTCCTGTAA |
| [ARFGEF1](http://probes.pw.usda.gov/batchprimer3/batch_primers/58.32.217.78_1449208657/58.32.217.78_14492086572.html) reverse | AGTCCACCGCATCTCTCTGT |
| ARHGAP39 forward  ARHGAP39 reverse  ATP6V1C1 forward  ATP6V1C1 reverse  BZW2 forward  BZW2 reverse  C11orf45 forward  C11orf45 reverse  C12orf11 forward  C12orf11 reverse  CCT2forward  CCT2reverse  CLEC2Dforward  CLEC2Dreverse  CMTM7forward  CMTM7reverse  CSPP1forward  CSPP1reverse  DCAF13forward  DCAF13reverse  DIMT1Lforward  DIMT1Lreverse  DKC1forward  DKC1reverse  DPH2forward  DPH2reverse  DSCC1forward  DSCC1reverse  FAM49Bforward  FAM49Breverse  FAM60Aforward  FAM60Areverse  GNL3forward  GNL3reverse  HEATR2forward  HEATR2reverse  IARSforward  IARSreverse  INTS8forward  INTS8reverse  KIAA0020forward  KIAA0020reverse  KIAA0196forward  KIAA0196reverse  MINAforward  MINAreverse  MKI67IPforward  MKI67IPreverse  MYCforward  MYCreverse  NBNforward  NBNreverse  NCLforward  NCLreverse  NSMAFforward  NSMAFreverse  NUDCD1forward  NUDCD1reverse  PABPC3forward  PABPC3reverse  PNO1forward  PNO1reverse  PUS7forward  PUS7reverse  RIPK2forward  RIPK2reverse  RRS1forward  RRS1reverse  SLC25A32forward  SLC25A32reverse  SLC38A1forward  SLC38A1reverse  SNHG4forward  SNHG4reverse  SSBforward  SSBreverse  TGS1forward  TGS1reverse  TMEM65forward  TMEM65reverse  TMEM67forward  TMEM67reverse  TRMT6forward  TRMT6reverse  UBE2E1forward  UBE2E1reverse  UTP23forward  UTP23reverse  WDR12forward  WDR12reverse  YWHAZforward  YWHAZreverse  ZFAND1forward  ZFAND1reverse  ZHX2forward  ZHX2reverse | CCCCAAGTTCCACTCCTACC  GATGCCCTCTGTCTGGTCA  GGATGTCTTGGTTGGCTTGT  CAGGGACTGCTTGATTGGAT  CACCAGTCTCTTCACCGACA  CATAAAGCACCACCTCCTTGA  CACCTGAGTAGCACGACCTC  CACCTGAGTAGCACGACCTC  CAAGCGGTAGTTCCATTAGCC  CCTCGTTTCTTTCGTTCTTCC  AGAACACCAGGCAAAGAAGC  GTCCACACGCAGAATCACC  GACACCAAGAACTGGACATCAA  TTCAAATAGGCACACTCTCCTG  GGACCAACTACAGCGCCTAC  TGCATCTGTGGACTGGGTTA  GGATGCTCTTTGGAGGTGAA  AGGATGAACTGATGGGATGG  CTTTTGAGGTCCCACGAGAA  CCATCCATTTTCCACTGCTT  GGGTGATGTGCTGAAAACAG  GGTGGTGGTGGATTCTTAGG  GGTCCAAAGGCAAGTCAGAA  ATCACTGTCCCCATCTCCAG  GGAGACGACAGGGTCAAAGA  CTCACTCAGCAGCACCACAG  TTGCTTTTCATTCCTGGTTG  TTTCTTCCTCACTTGCCTGA  TATTCTCCCACCCAGCATCT  TCAAGGTTTTCAGCATTGGA  CACAAGCCAAAGATGTACCG  GCTTTTTATCCTGTTCCCAGA  TGACCTGCCATAAGCGGTAT  AGCCCAAACTCCTTTTCCAT  GACAAGGCACAGGAGACGAT  GGTGGACAGGATGGAGAACA  GTTTGTGCGAGTGGAGAACA  TGAAGGAATGGTCAGGTGGT  CCAGTCAAACAGGCAAAACC  GCCCAAATCATAGCACACCT  GCGGCATTTGATTGTATTGA  CACTTCTTGGGCGTGTTCTT  CTTTCTTTCGCTTGGCAGTT  GCGGGTAGGCACTTCAATAA  AATCTCGCCCATCAAGACAG  CTGAATCGTTGCCCTTTTCT  CGGAATCGGACACTAACACA  TTTCGCCTCTCCAAAAATG  CCGAGGAGAATGTCAAGAGG  ACGCACAAGAGTTCCGTAGC  AGAATGGCTTTTCCCGAACT  GGCTGCTTCTTGGACTCAAC  CCAGCCATCCAAAACTCTGT  TCTACCACCACCTCGTCCTC  AGAAAGGGGCAAAATGGAAT  CGTTGAGGGGCTGAAAATAC  AAATGCCCTGCTTCTGTTTG  AGGCTTGCTACTTGCTGCTT  TTCACAGGTTCCACGAGTCA  TTTTCCCAGCAAGAGTAGGG  CATTTGGGACTTCAGATACGC  TTTGGTTTTTCCTCCTTTGC  GTAATGCCCTTGCCTGGTT  GAGCCCTGTATTTGCCTTCA  AAAGGGCTGCATTCTGTGAT  TGGCAAATTCTTCTCCTTGG  ACAACACGCAACTGCTCATC  GCACCTCAATCAGCCATTCT  TTGATGGACTACGGGGACTT  TATTGTCGGTGTGGGGAGTT  GGTGTTATTTTTCACGGTTCG  GGAATGCTGACCAAGGAGAA  AGATGGGGGTAGGGGACTTA  CAAATCGCAAGGTCAGGAGT  GCAAACCCCTACCTGAAGTG  TGGCAAAGTAATCGTCCTTG  GGAACCAATGAGGAAAGCAA  GACCTGCCGATGACTGAAAT  CCACAATGCGATACCTTTCA  CAGCCAATAGTCACCCCAAC  TTGAAGACAGCAGGATGGAA  CTTTAGGTCGCTCCCAATCA  TAGTCGTTTCCACCCCACTC  ATTTGAGGCTGGTGTCTGCT  TCCCAAAGGCGATAACATCT  AAGGGGTCGGCAGGATTA  TGCACAAAAATGCCAAGTTC  TGCACTGAGACAAGCTGACC  TCCCTTCTCAATCCCTGCT  CCATTCCTCTGCCCCTTTA  AAAGACGGAAGGTGCTGAGA  TCTGATAGGATGTGTTGGTTGC  TTGAACACAGAAGCAGGGAGT  GGCACCTTTCCATCGTTTAC  AAGAGAAAGGAATCGGCACA  TGGTCGGATAGGGAGTCGTA |
